# Redox-Sensitive Linear and Cross-Linked Cystamine-Based Polymers for Colon-Targeted Drug Delivery: Design, Synthesis, and Characterisation

**DOI:** 10.3390/pharmaceutics12050461

**Published:** 2020-05-18

**Authors:** Yoke Mooi Ng, Siti Nur Aishah Mat Yusuf, Hock Ing Chiu, Vuanghao Lim

**Affiliations:** 1 Integrative Medicine Cluster, Advanced Medical and Dental Institute, Universiti Sains Malaysia, Bertam, Kepala Batas 13200, Penang, Malaysia; yumei.wu91@gmail.com (Y.M.N.); nuraishahyusuf@unimap.edu.my (S.N.A.M.Y.); hockingchiu@gmail.com (H.I.C.); 2 Department of Chemical Engineering Technology, Faculty of Engineering Technology, UniCITI Alam Campus, Universiti Malaysia Perlis, Padang Besar 02100, Perlis, Malaysia

**Keywords:** redox-sensitive, linear, crosslinked, disulfide, thiol, cystamine, cysteamine

## Abstract

Cystamine-based polymers may help to achieve controlled and targeted drug delivery to the colon due to their susceptibility to breakage of the disulfide linkage in the low redox potential environment of the colon. In this study, two linear cystamine-based polymers with similar repeating units (LP1 and LP2) and a cross-linked cystamine-based polymer (BP) were synthesised and their kinetics and the various physical conditions underlying cystamine-based polymerisation were evaluated. In brief, *N*^1^,*N*^6^-bis(2-(tritylthio)ethyl)adipamide (**2**) was synthesised from the reaction of triphenylmethanol and cysteamine. Next, the trityl group of **2** was removed with trifluoroacetic acid and triethylsilane before proceeding to oxidative polymerisation of the end product, *N*^1^,*N*^6^-bis(2-mercaptoethyl)adipamide (**3**) to LP1. The Schotten-Bauman reaction was applied to synthesise LP2 and BP from the reaction of cystamine with adipoyl chloride or trimesoyl chloride. Scanning electron microscopy, energy-dispersive X-ray spectroscopy, and mapping showed that oxygen, nitrogen, sulfur, and carbon were homogenously distributed in the polymers, with LP2 and BP having less porous morphologies compared to LP1. Results of zinc-acetic acid reduction showed that all polymers began to reduce after 15 min. Moreover, all synthesised polymers resisted stomach and small intestine conditions and only degraded in the presence of bacteria in the colon environment. Thus, these polymers have great potential for drug delivery applications. LP2 and BP, which were synthesised using the Schotten-Bauman reaction, were more promising than LP1 for colon-targeted drug delivery.

## 1. Introduction

Over the past two decades, colon-targeted drug delivery systems have been widely investigated for the treatment of inflammatory bowel disease, colon cancer, and circadian rhythm type diseases [1,2]. Regardless of the therapeutic strategy, the oral route is still considered to be the preferable choice for colon-targeted drug delivery compared to other modes of drug administration [3]. However, the delivery of protein or peptide drugs is challenging as the amount of the drug(s) that actually reach(es) the colon after oral ingestion is low due to the harsh environment of the stomach and upper bowel region (i.e., acidic environment and presence of proteolytic enzymes) [1,4].

This problem can be resolved by developing stimuli-responsive polymeric coatings for drugs such that the coating will break down only when exposed to endogenous stimuli unique to the colon, such as a specific pH value, microflora mixture, and redox potential [4,5]. Polymer coatings with time dependent release systems are also one of the strategies for colon targeted drug delivery [1]. Various drug carriers or coating systems that are sensitive to pH changes (such as Eudragits^®^, Asacol^®^, and etc) and transit time dependent such as Pulsincap, or even the combination of both (dual responsive stimuli) are commercially available [6,7,8]. Such polymers act as protective drug carriers that could safely pass through the harsh environment of the upper gastrointestinal tract and subsequently release the drug only when specific conditions at the targeted site were met. Yet, these stimuli-based systems may have the possibility of premature drug release in the small intestine. The transit time is difficult to estimate because gastric emptying time is influenced by the dietary behaviour of each individual and the differences in pH values from small intestine to colon. Such issues had resulted in poor site-specific drug delivery [9]. On the other hand, redox-responsive polymers are currently favoured by researchers, as the colon has the lowest redox potential of all gastrointestinal regions. The azo polymer is one of the redox-responsive polymers that has been widely studied for colon-targeted drug delivery [10,11]. However, this polymer has disadvantages, such as toxic by-products, aromatic amines and hydrazo compounds, and its rate of degradation is low due to poor water solubility of the azo linkage [12].

According to the literature, disulfide-based polymers similar to the azo polymer are highly reducible in the colon environment and remain fairly intact in the acidic and enzymatic environments of the stomach and small intestine; thus, they may effectively serve as an alternative to the azo polymer [13,14,15,16]. Disulfide-containing polymers have been widely explored and applied in intracellular-targeted drug delivery systems [17], but their potential as a coating material for colon-targeted drug delivery has received little attention. Furthermore, whether linear or cross-linked polymers would provide a better degradation profile during colon-targeted drug delivery remains unexplored. The current understanding is that linear disulfide polymers would have high solubility and could therefore be degraded under low pH conditions [18], whereas cross-linked disulfide polymers with low solubility would have a poorer degradation profile.

In the present study, two linear and one cross-linked disulfide polymers were synthesised using hydrophilic cystamine and cysteamine, and then their chemical and degradation properties were evaluated. Cystamine is a disulfide diamine compound generated from the oxidation of two cysteamine thiols [19]. Both cystamine and cysteamine are found within the human body, thus potentially suitable for pharmaceutical purposes and as cross-linkers for redox function in drug carriers [20,21].

## 2. Materials and Methods

### 2.1. Materials

2-aminoethane, 5,5′-dithio-bis-(2-nitrobenzoic acid) (Ellman’s reagent), adipoyl chloride, cystamine dihydrochloride, dialysis tubing cellulose membrane (MWCO 12,000 Da), dimethylsulfoxide (DMSO), dimethylsulfoxide-d_6_ (DMSO-d_6_), pancreatin, pepsin polyvinyl alcohol (PVA), sodium nitroprusside, triethylsilane (TES), trifluoroacetic acid (TFA), trimesoyl chloride, triphenylmethanol, Tween 80, and zinc dust were purchased from Sigma Chemicals (St. Loius, Missouri, US). Ammonium bicarbonate, calcium chloride (CaCl_2_), dichloromethane (DCM), diethyl ether (Et_2_O), disodium hydrogen phosphate (Na_2_HPO_4_.2H_2_O), hydrochloric acid (HCl), monopotassium phosphate (KH_2_PO_4_), sodium bicarbonate (NaHCO_3_), sodium chloride (NaCl), sodium hydroxide (NaOH), and sulfuric acid (H_2_SO_4_) were purchased from R&M Chemicals (London, UK). Silica gel (0.035–0.070 mm) was purchased from Acros Organics (Geel, Belgium) and used for column chromatography. Acetic acid and methanol were purchased from Qrec Chemical (Chonburi, Thailand). *Lactobacillus* was provided by the Department of Biological Science and Technology, China Medical University, Taichung, Taiwan.

### 2.2. Preparation of Cysteamine-Based Dithiol Monomer

#### 2.2.1. Synthesis of (triphenylmethyl)thioethylamine, **1**

**1** was synthesised according to the procedure reported by Lim et al. [22]. In a round bottom flask, 2-aminoethane (2.272 g, 0.020 mol) was added and dissolved in TFA (50 mL) with constant stirring, followed by the addition of triphenylmethanol (5.207 g, 0.020 mol) for 3 h. To keep moisture out of the reaction, an L-shaped connector containing CaCl_2_ was prepared and used to close the round bottom flask. A rotary evaporator was used to remove the TFA, and the remaining thick brown oil was washed with a volume of anhydrous diethyl ether to form the white precipitate. The precipitate was filtered, dried, dissolved with anhydrous diethyl ether, and partitioned with 1 M NaOH. The diethyl ether portion was collected and evaporated off using the rotary evaporator to yield a white precipitate with the following characteristics based on various analyses: IR (KBr disk, cm^−1^): 3356, 3321 (primary –N–H stretching), 2920 (–C–H stretching), 1753–1952 (aromatic overtone), 1589 (–N–H bending); ^1^H-NMR (DMSO-d_6_, δ): 7.23–7.34 ppm (15 H, m, Ar-H), 2.40–2.43 ppm (2 H, t, *J* = 5 Hz, CH_2_–NH_2_), 2.14–2.17 ppm (2 H, t, *J* = 5 Hz, S–CH_2_); DEPTQ (DMSO-d_6_, δ): 145.14 (3 C, Ar–C, negative phase), 129.57 (6 C, Ar–C, positive phase), 128.44 (6 C, Ar–C, positive phase), 127.11 (3 C, Ar–C, positive phase), 66.22 (1 C, S–C–Ar_3_, negative phase), 41.31 (1 C, CH_2_–NH_2_, negative phase), 36.12 (1 C, S–CH_2_, negative phase) (Figures S1–S3 in Supplementary Materials); LC–MS: *m*/*z* [M + H]^+^ 320.46.

#### 2.2.2. Synthesis of *N*^1^, *N*^6^-bis(2-(tritylthio)ethyl)adipamide, **2**

The synthesised **1** (3.190 g, 10 mmol) was dissolved in NaOH basic solution (0.2 M, 50 mL) with constant stirring in a round bottom flask and heated at 35 °C. Next, adipoyl chloride (0.72 mL, 5 mmol) solution was prepared in 50 mL of anhydrous DCM and added dropwise into the mixture at the speed of 700 rpm. The mixture was refluxed for 16 h and cooled at room temperature. The resulting white precipitate was filtered, dried, and dissolved in anhydrous DCM, then washed with 1 M H_2_SO_4_, 1 M NaHCO_3_, and 1 M NaCl following the liquid-liquid extraction method. The DCM phase was collected and solvent was removed using rotary evaporator. Purification of **2** was conducted using column chromatography with a mobile phase of Et_2_O: DCM = 6:4. The compound had the following characteristics: IR (KBr disk, cm^−1^): 3278 (secondary –N–H stretching), 2982 (–C–H stretching), 1774–1956 (aromatic overtone), 1640 (C=O), 1554 (–N–H bending); ^1^H-NMR (DMSO-d_6_, δ): 7.85–7.87 (2 H, t, *J* = 5 Hz, C=ONH), 7.23–7.35 ppm (15 H, m, Ar–H), 2.94–2.98 (4 H, q, *J* = 7 Hz, CH_2_–NH), 2.16–2.19 (4 H, t, *J* = 7 Hz, S–CH_2_), 1.98 ppm (4 H, s, CH_2_–C=O), 1.39 ppm (4H, s, CH_2_CH_2_–C=O); DEPTQ (DMSO-d_6_, δ): 172.37 (2 C, C=ONH, negative phase), 144.87 (3 C, Ar–C, negative phase), 129.50 (6 C, Ar–C, positive phase), 128.51 (6 C, Ar–C, positive phase), 127.21 (3 C, Ar–C, positive phase), 66.35 (1 C, S–C–Ar_3_, negative phase), 37.84 (2 C, CH_2_–NH, negative phase), 35.50 (2 C, O=C–CH_2_, negative phase), 31.93 (2 C, CH_2_CH_2_–C=O, negative phase), 25.34 (S–CH_2_, 2 C, negative phase) (Figures S4–S6 in Supplementary Materials); LC–MS: *m*/*z* [M + H]^+^ 750.03.

#### 2.2.3. Removal of Trityl Group, *N*^1^, *N*^6^-bis(2-mercaptoethyl)adipamide, **3**

The detritylation method was adapted from Pearson et al. [23] with modifications. Compound **2** (0.4 g) was dissolved in DCM in a round bottom flask. TFA (0.863 mL) was added dropwise to the solution while stirring followed by TES (0.5 mL) for 5 h at room temperature. The solvent was removed using the rotary evaporator to yield a white precipitate (**3**). The precipitate was washed with copious anhydrous diethyl ether, filtered, dried in vacuo over silica gel, and analysed using various methods: IR (KBr disk, cm^−1^): 3296 (secondary N–H stretching), 2982 (–C–H stretching), 2545 (–S–H), 1640 (–C=O) 1545 (–N–H, bending); ^1^H-NMR (DMSO-d_6_, δ): 7.95–7.97 (2 H, t, *J* = 5 Hz, C=ONH), 3.16–3.20 (4 H, q, *J* = 7 Hz, CH_2_–NH), 2.04–2.07 (4 H, t, *J* = 6 Hz, CH_2_C=O), 1.44–1.47 ppm (4 H, q, *J* = 4 Hz, CH_2_CH_2_–C=O); DEPTQ (DMSO-d_6_, δ): 172.51 ppm (2 C, C=O, negative phase), 42.50 (2 C, CH_2_–NH, negative phase), 35.62 (2 C, CH_2_–C=O, negative phase), 25.42 (2 C, CH_2_–SH, negative phase), 23.99 (2 C, CH_2_CH_2_–C=O, negative phase) (Figures S7–S9 in Supplementary Materials); LC–MS: *m*/*z* [M + H]^+^ 265.41.

### 2.3. Synthesis of Cystamine-Based Polymer via Oxidative Polymerisation, LP1

Compound **3** was stirred in ammonium bicarbonate buffer (0.1 M, pH 8.3), then DMSO was added dropwise until approximately half of the solid was dissolved [22]. The reaction was stirred in open air for 24–48 h until no more thiol could be detected with sodium nitroprusside reagent in the nitroprusside test, which was conducted as follows: An aliquot of the sample was added into ammonium bicarbonate (pH 8.3, 1 mL) solution. A few drops of 5% *w/v* of sodium nitroprusside aqueous solution were added to the prepared sample solution. The colour turns from colourless to pink-violet in the presence of thiol [24].

The white precipitate that yielded from this reaction was filtered, washed with water and methanol, then dried in vacuo over silica gel. The compound had the following characteristics: IR (KBr disk, cm^−1^): 3300 (secondary –N–H stretching), 2945 (–C–H stretching) 1638 (–C=O), 1541 (–N–H bending); ^13^C CP/MAS NMR (solid state, δ): 174.09 (C=ONH), 42.61 (CH_2_–NH), 39.24 (CH_2_–SS), 36.11 (CH_2_C=ONH), 25.62 (CH_2_–CH_2_) (Figure S10 in Supplementary Materials).

### 2.4. Alternative Method of Synthesising Cystamine-Based Linear Disulfide Polymer, LP2

Cystamine dihydrochloride (1.126 g, 0.005 mol) was dissolved in 50 mL of NaOH (0.4 g, 0.2 M) solution with constant stirring. Adipoyl chloride (1.144 mL, 0.005 mol) then was dissolved in 25 mL of DCM in a 100 mL beaker and added dropwise into the cystamine dihydrochloride solution. The resulting mixture was stirred and refluxed at 38 °C for 16 h. The obtained white precipitate was filtered and washed with distilled water and DCM, then dried in vacuo over silica gel. The compound had the following characteristics: IR (KBr disk, cm^−1^): 3300 (secondary –N–H stretching), 2945 (–C–H stretching) 1640 (–C=O), 1541 (–N–H bending); ^13^C CP/MAS NMR (solid state, δ): 174.58 (C=ONH), 42.70 (CH_2_–NH), 39.29 (CH_2_–SS), 35.98 (CH_2_C=ONH), 25.73 (CH_2_–CH_2_) (Figure S11 in Supplementary Materials).

### 2.5. Synthesis of Cystamine-Based Crosslinked Polymer, BP

The synthesis procedures were adapted from Broaders et al. [25] with some modifications. Briefly, cystamine dihydrochloride (0.677 g, 0.003 mol) was dissolved in 50 mL of 0.2 M NaOH solution in a beaker. Next, 25 mL of trimesoyl chloride solution (0.265 g, 0.001 mol, prepared in DCM) were added dropwise into the cystamine dihydrochloride solution. The reaction was stirred for 5 h. The resulting precipitate was filtered and washed with distilled water and DCM, then dried in vacuo over silica gel. The compound had the following characteristics: IR (KBr disk, cm^−1^): 3299 (secondary –N–H stretching), 2930 (–C–H stretching) 1648 (–C=O), 1536 (–N–H bending); ^13^C CP/MAS NMR (solid state, δ): 167.0 (C=ONH), 134.0 (Ar–C), 40.17 (CH_2_–NH) (Figure S12 in Supplementary Materials).

### 2.6. Physical Characterisation of the Synthesised Compounds

#### 2.6.1. Fourier Transform Infrared Resonance (FTIR)

FTIR spectra of the synthesised compounds were recorded from potassium bromide disks using a Nexus 670 FTIR (Thermo Nicolet, Madison, WI, USA) with wavelength range from 500 to 4000 cm^−1^ [13].

#### 2.6.2. 1-Dimensional Nuclear Magnetic Resonance (1D-NMR)

Samples of compounds **1**, **2**, and **3** were prepared in DMSO-*d_6_* and analysed using a Bruker Advance 500 device (Bruker, Billerica, MA, USA) at room temperature. ^1^H-NMR spectra were recorded at 500 MHz, whereas DEPT-Q spectra were recorded at 125 MHz.

#### 2.6.3. Solid State Nuclear Magnetic Resonance (CP/MAS ^13^C NMR)

A Bruker Advance III 400 NMR spectrometer (Bruker, Billerica, MA, USA) at 100 MHz was used to record the solid-state CP/MAS ^13^C NMR spectra of the synthesised polymers.

#### 2.6.4. Solubility Test for Disulfide Cross-Linked Polymers

Solubility test for disulfide cross-linked polymers were carried out according to Lau and Lim [18] with slight modification. Briefly, various types of organic solvents such as trifluoroacetic acid, acetic acid, formic acid, acetone, chloroform, cyclohexane, dichloromethane, dimethylsulfoxide, ethanol, toluene, water and buffer system of simulated gastric (without pepsin), intestinal (without pancreatin) and colon (without *Lactobacillus*) were used for the solubility test. 3 mg of polymer (LP1, LP2, BP) was inserted into an Eppendorf tube. 1 mL of DCM was later added into the tube and the mixture was spinned for 5 min using a homogeniser. The mixture was then observed under the bright light to determine the solubility of the polymer. These steps were repeated for different organic solvents and phosphate buffers with different polymers.

#### 2.6.5. Melting Point Analysis

The melting point of synthesised compounds **1**, **2**, and **3** was measured using a MP10 melting point analyser (Stuart, Staffordshire, UK). The sample was first placed in a capillary tube, which was placed in the melting point apparatus prior to heating. The temperature was recorded when the sample began to melt and when it was completely molten.

#### 2.6.6. Scanning Electron Microscopy-Energy Dispersion X-Ray (SEM-EDX)

The surface morphologies of the synthesised polymers were analysed using SEM. The sample was first coated with gold using a sputter coater, then viewed under the SEM. A detection-microanalysis-system (INCA 400, Oxford Instruments PLC, Bucks, UK) was applied for EDX analysis of the polymers.

### 2.7. Chemical Reduction Studies of the Synthesised Disulfide Polymers

#### 2.7.1. Measurement of Thiol Using Ellman’s Reagent

The protocol was based on Mat Yusuf et al. [26] with slight modification. Ellman’s reagent (0.1 M) was prepared in pH 7.4 phosphate buffer containing EDTA (1 mM). To each test tube, 50 μL of Ellman’s reagent and 2.5 mL of pH 7.4 phosphate buffer were added, followed by 250 μL of standard or unknown. For the blank, 250 μL of pH 7.4 phosphate buffer were added instead of thiol-containing solution. All solutions were mixed well and incubated at room temperature for 15 min. An absorbance reading was taken for each unknown and standard in a 1 cm cell quartz cuvette using a UV-visible spectrophotometer (Perkin-Elmer Lambda 25, Madison, WI, USA) set to 412 nm. The concentration of thiol in each unknown and standard was obtained by applying the Beer-Lambert equation:

$$\varepsilon =\frac{A}{cd}$$

where *c* = concentration (mol/L), *A* = absorbance, ε = molar attenuation coefficient at 412 nm (14,150 M^−1^ cm^−1^), and *d* = cell pathlength (1 cm).

As dilution was conducted earlier in the procedure, the dilution factor was accounted for as follows:

$$\mathrm{Concentration}\;\mathrm{of}\;\mathrm{unknown}/\mathrm{standard},\;\mathrm{mol}/L\;=\frac{A}{\varepsilon d}\times \frac{\mathrm{total}\;\mathrm{volume}\;\mathrm{in}\;a\;\mathrm{test}\;\mathrm{tube}\;\left(L\right)}{\mathrm{volume}\;\mathrm{from}\;\mathrm{unknown}\;\mathrm{sample}\;\left(L\right)}$$

#### 2.7.2. Chemical Reduction Studies

Cystamine dihydrochloride (0.5 g, 2.2 mmol) and acetic acid (1.3 mL) were dissolved in distilled water (10 mL) in a 3-neck round bottom flask. The mixture was purged with nitrogen for 15 min and refluxed at 100 °C. Zinc dust (1.950 g) was then slowly added while stirring at slow speed. The mixture (10 μL) was withdrawn from the side arm of the round bottom flask and diluted to 25 mL with distilled water. A Pasteur pipette with pre-inserted cotton wool was used to filter the diluted sample solution. To estimate the free thiol content, 250 μL of filtrate was used to conduct the Ellman’s test. The procedure was repeated by replacing the cystamine dihydrochloride with 2 g of polymer (LP1, LP2, and BP).

### 2.8. Degradation Studies of Disulfide Polymers

In vitro degradation of disulfide polymer studies were carried out according to Lau and Lim [18] with slight modification. Each polymer was run in triplicate.

#### 2.8.1. Preparation of de-Aerated SØrensen Phosphate Buffer, pH 7.4

SØrensen phosphate buffer at pH 7.4 was prepared by mixing 0.1 M of Na_2_HPO_4_.2H_2_O with 0.1 M KH_2_PO_4_. The buffer solution was autoclaved for 20 min (121 °C, 0.12 MPa) and then stirred at low speed while heating to 41 °C. The buffer was immediately filtered using a filter paper with porosity size of 0.45 µm under vacuum with vigorous stirring in the conical flask. The stirring process was continued for another 30 min under vacuum until no more bubbles were seen [27]. The buffer was then transferred into a Schott bottle, sealed, and stored in the refrigerator until further use. Prior to use, the de-aerated buffer was heated to 37 °C while purging with oxygen-free nitrogen for 1 h.

#### 2.8.2. Preparation of Polymers in Visking Dialysis Bags

A visking dialysis tube (20 cm) was cut and softened in warm distilled water. One end of the tube was tightened to form an open sac on the other side before loading the polymer (4 g).

#### 2.8.3. Incubation of Polymers in Simulated Gastric Fluid

Visking dialysis tubes that initially were filled with 4 g of polymer were placed in a pre-warmed (37 °C) Erlenmeyer flask containing 50 mL of pH 1.2 simulated gastric fluid (0.32 % of pepsin and 2 mg/mL of NaCl in 0.1 M HCl) [27]. The incubation was continued for 2 h in a shaking water bath at 50 rpm and temperature of 37 °C. Degradation of the disulfide polymers was evaluated by withdrawing a sample every 30 min and measuring its thiol concentration using the Ellman’s reagent test. For every 250 µL sample taken, 250 µL of simulated gastric fluid were added to the sample reaction mixture.

#### 2.8.4. Incubation of Polymers in Simulated Intestinal Fluid

The visking dialysis tubes from the previous section were placed in a pre-warmed (37 °C) Erlenmeyer flask containing 50 mL of simulated intestinal fluid (1.0% pancreatin in pH 6.8 phosphate buffer) [27]. The incubation was continued for 3 h in a water bath at 50 rpm and temperature of 37 °C. Degradation of the disulfide polymers was evaluated by withdrawing a sample every 30 min and measuring its thiol concentration using the Ellman’s reagent test. For every 250 µL sample taken, 250 µL of simulated intestinal fluid were added to the sample reaction mixture.

#### 2.8.5. Incubation of Polymers with Lactobacillus in SØrensen’s Phosphate Buffer

*Lactobacillus* pellet was inserted into the visking dialysis tubes it. The opened end of the visking dialysis bag was then closed by tying a knot, and the bag was placed in 50 mL of anhydrous SØrensen’s phosphate buffer in an Erlenmenyer flask. The Erlenmenyer flask was sealed with a rubber sheet and continuously flushed with oxygen-free nitrogen through a sterile needle. The incubation process was continued for 36 h in a shaking water bath at 50 rpm and temperature of 37 °C. Degradation of disulfide polymers was evaluated by withdrawing a sample at pre-set time intervals and measuring its thiol concentration using the Ellman’s reagent test. For every 250 µL sample taken, 250 µL of pre-flushed oxygen-free nitrogen SØrensen’s phosphate buffer were added to the sample reaction mixture.

#### 2.8.6. Control Incubation

Two sets of control incubations in simulated colon conditions were conducted. The first control contained the polymer incubated alone in SØrensen’s phosphate buffer without the presence of *Lactobacillus*, and the second control contained *Lactobacillus* alone incubated in de-aerated SØrensen’s phosphate buffer.

#### 2.8.7. Statistical Analysis

Statistical analysis using IBM SPSS software Version 22 (Armonk, NY, USA) was carried out for the following data: final thiol concentration at hour 2 of the simulated gastric condition, hour 3 of the intestinal condition, and hour 32 of the simulated colon condition for the different polymers. Final thiol concentration of incubated polymers with bacteria, bacteria only, and polymer only in the simulated colon condition also were tested. The data were first analysed using one-way analysis of variance to test for homogeneity of variance at *p* < 0.05 so to determine either Dunnet’s 2-side or Dunnet’s T3 to be used for later post-hoc testing.

## 3. Results and Discussion

### 3.1. Synthesis and Characterisation of 3

**Scheme 1:**
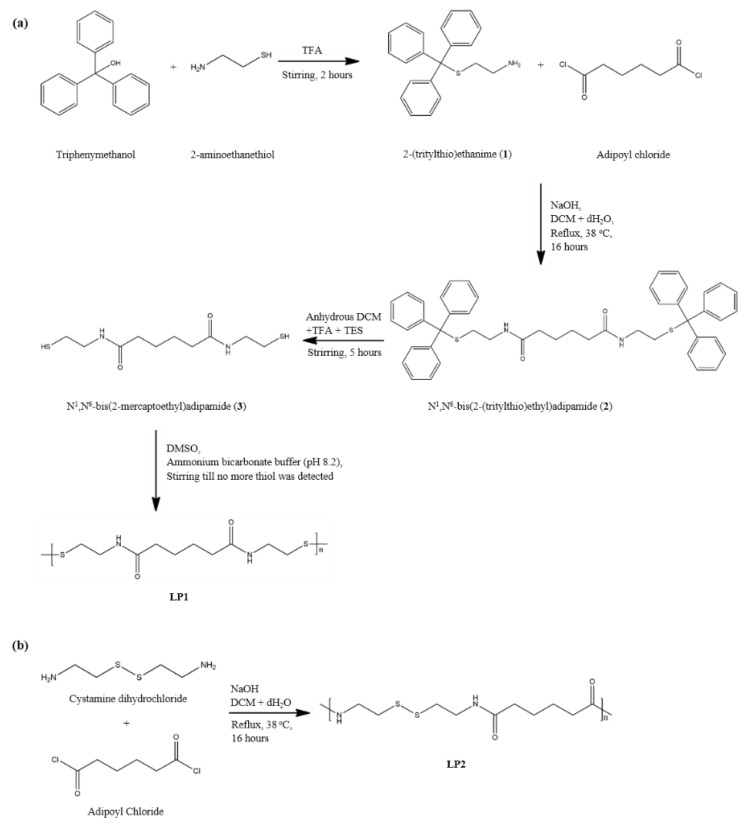
The synthetic routes, reagents, and conditions for synthesis of (**a**) LP1 and (**b**) LP2.

**Figure 1:**
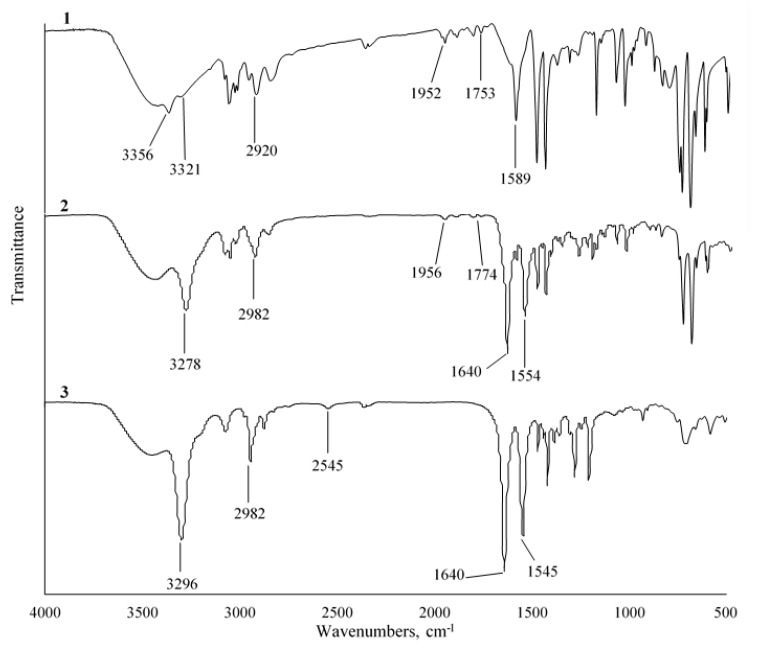
FTIR spectra of compounds **1**, **2**, and **3**.

Dithiol monomer **3** was obtained as shown in Scheme 1a. First, **1** was synthesised from the reaction of triphenylmethanol and 2-aminoethane thiol. Liquid-liquid extraction with NaOH was performed to remove any excessive residue of TFA to yield a white residue. The percentage yield was recorded at 90–95% with melting point of 92–96 °C. Subsequent TLC analysis showed band 1 at R_f_ 0.33 using the solvent system of ethanol:petroleum ether = 1:1, *v/v*. Success of the reaction that yielded compound **1** was illustrated by aromatic bending overtone peaks in the region of 1753–1952 cm^−1^ in its IR spectrum (Figure 1). It was also supported by the absence of thiol peak in the spectra as well as the acquisition of a negative outcome from the nitroprusside test. The successful synthesis of compound 1 was also shown by the presence of two multiples in the regions of 7.23–7.26 ppm and 7.32–7.34 ppm, which were ascribed to aromatic protons (Ar–H) from the trityl group. The presence of the trityl group in compound **1** was further confirmed by its DEPTQ spectra, in which the 18 aromatic carbons from the trityl group were denoted respectively at 127.11, 128.44 and 129.57, and 145.15 ppm in the spectrum.

Subsequently, the reflux process was performed to synthesise the cystamine-based protected monomer **2** from the reaction of **1** and adipoyl chloride in basic solution. A white precipitate was obtained immediately upon the addition of the adipoyl chloride to the basic solution of 1. The pure compound **2** was obtained from column chromatography with R_f_ at 0.68. The percentage obtained yield was recorded at 61–65% with melting point of 184–187 °C. In the FTIR spectrum of 2 (Figure 1), the bending of *v* (N–H) exhibited a negative shift from the previous 1589 cm^−1^ to 1554 cm^−1^, and a new peak appeared at 1640 cm^−1^ that was attributed to *v* (C=O). These results indicate the involvement of N-H in the reaction and the subsequent conversion to secondary amide upon the reaction between the amine bond of **1** with adipoyl chloride [28]. Furthermore, C=ONH and C=ONH were assigned to the respective new triplet peaks at 7.85–7.87 ppm in the ^1^H-NMR spectrum and 172.38 ppm in the downfield DEPTQ spectrum of **2**. These results were further validated the successful synthesis of 2.

Next, the protected trityl group of **2** was removed using TFA with the addition of a scavenger, TES, to yield the final product, **3**. The solution turned yellowish upon the addition of TFA but reverted back to being colourless with the subsequent addition of TES. This occurred because the trityl cation presents as yellowish and addition of TES prevents the reverted reaction by reducing it to colourless triphenylmethane solution [23,29]. The percentage yield of white powder 3 was 83–87% with an observed melting point of 142–148 °C. The yield was further evaluated using the qualitative nitroprusside test, in which the colourless solution turned violet purple in colour, indicating the presence of sulfhydryl [30]. Figure 1 shows the disappearance of aromatic *v* (C–H) overtone bending and the appearance of a new peak at 2545 cm^−1^ in the FTIR spectrum, which is attributed to the *v* (S–H). This illustrated that removal of the trityl group from **2** to form **3** had occurred. This was further confirmed by the absence of aromatic protons and carbons in the ^1^H-NMR and DEPTQ spectra of **3**. Upon the removal of trityl groups, no more tertiary carbons were available in the structure of **3**, hence all carbon peaks were observed to have emerged at the negative phase in the DEPTQ spectrum.

### 3.2. Synthesis and Characterisation of Polymers

**Scheme 2:**
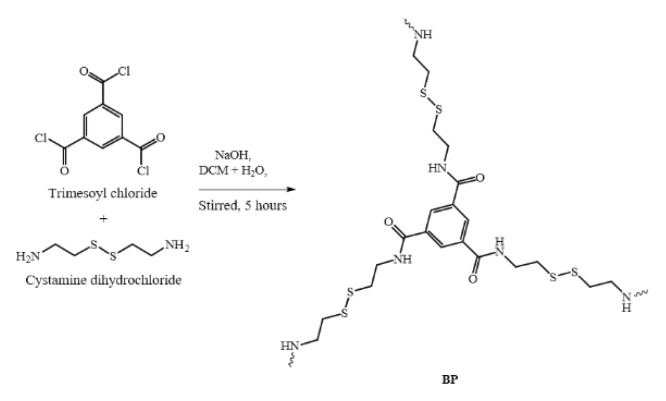
Synthetic routes, reagents, and condition for synthesis of BP.

As shown in Scheme 1a, LP1 was synthesised through oxidative polymerisation of **3** with a mild oxidising agent, DMSO [31], in ammonium bicarbonate buffer, then a white precipitate was recovered at the end of the reaction. The rate of oxidisation will be slow with air alone as oxidising agent and without the aids from polar aprotic solvent [32]. Thus, DMSO was chosen as the oxidising agent because it was highly stable throughout the reaction, did not form any notable side reactions, and was able to act as a solvent for the monomer [33,34,35]. The ammonium bicarbonate buffer functioned as a base and catalyst to help enhance the oxidation of thiols to disulfide in the reaction [36,37]. A negative result was observed for the nitroprusside test, which suggested that no thiol was present and that LP1 was successfully synthesised. The second cystamine-based linear polymer (LP2) was synthesised through the Schotten-Baumann acylation reaction to form amide bonding between adipoyl chloride and cystamine dihydrochloride (Scheme 1b). Formation of white precipitate was immediately observed when the adipoyl chloride was added dropwise into the cystamine solution in the presence of NaOH. The role of the NaOH in this reaction was to trap the side product HCl and prevent it from forming salt with cystamine that would reduce the yield of LP2 [38]. Subsequently, similar reaction concept of LP2 was performed to obtain cystamine based cross-linked polymer (BP) in the presence of NaOH basic solution (Scheme 2). Therefore, similar observations were observed for LP2 and BP throughout the reaction.

#### 3.2.1. FTIR Analysis

**Figure 2:**
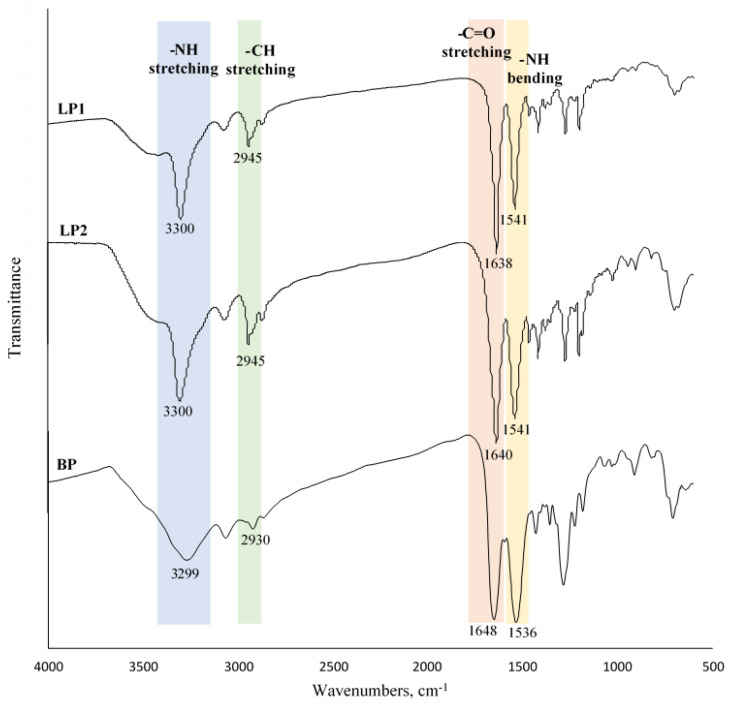
FTIR spectra of LP1, LP2, and BP.

Free thiol *v* (S–H) was not detected in the IR spectrum of LP1 (Figure 2), which suggested that the polymerisation of **3** by oxidising the free thiol to form LP1 was successful. LP2 was synthesised by formation of an amide bond from cystamine dihydrochloride and adipoyl chloride. Evidence for the successful formation of LP2 is in the appearance of the bond stretching of secondary amide *v* (N–H) and carbonyl *v* (C=O), which were found at 3300 and 1638 cm^−1^, respectively, in the IR spectra (Figure 2).

Meanwhile, due to BP being produced by similar Schotten Bauman acylation reaction for that of LP2, the observation of *v* (N–H) at 3299 cm^−1^ (stretching) and 1536 cm^−1^ (bending), and *v* (C=O) at 1648 cm^−1^ amide characteristic peaks in IR spectra were systematically assigned for BP (Figure 2). The shoulder peak at 1700–1750 cm^−1^ that would indicate the presence of trimesoyl chloride was absent in the IR spectra, which indicates that no unreacted trimesoyl chloride was present due to the involvement of it in amide bond formation [28,39].

#### 3.2.2. NMR Analysis

In the ^13^C CP/MAS NMR spectra of LP1 and LP2, the C=ONH peak could be resolved at 174.09 (LP1) and 174.58 ppm (LP2). Furthermore, the CH_2_–NH peak of LP1 and LP2 could also be assigned to 42.61 and 42.70 ppm, respectively. These assignments were in full agreement with results reported by Mathias and Johnson [40] and Hatfield et al. [41] for their nylon synthesis. Moreover, the stretching and bending of secondary amide *v* (N–H) as well as *v* (C=O) in the IR spectra of LP1 and LP2 (Figure 2) further reinforced the premise of complete formation of LP1 and LP2. Even though LP1 and LP2 were synthesised using different methods, both polymers exhibited common characteristic signals at the same vibrational frequency in the IR spectra and chemical shifts in the ^13^C CP/MAS NMR spectra due to their similar repeating units.

The ^13^C CP/MAS NMR spectrum of BP differed from those of LP1 and LP2, as BP had a broad signal at 134.0 ppm that represented the aromatic-C. Similarly, Williams et al. [39] previously reported that their polyamide aerogel had an aromatic carbon centred at 137.0 ppm. The C=ONH signal of BP was assigned to 167.0 ppm [42] and was in tandem with the observed *v* (C=O) in the IR spectrum (Figure 2) centred at 1648 cm^−1^, which demonstrated successful synthesis of the amide linkage in BP.

#### 3.2.3. Solubility of LP1, LP2, and BP

Solvents of different polarity were applied to evaluate the solubility of LP1, LP2, and BP at room temperature (Table 1). Although LP1, LP2, and BP are different forms of polymers, all three had similar solubility (i.e., good in formic acid and trifluoroacetic acid).

#### 3.2.4. SEM Morphology of the Polymers

**Figure 3:**
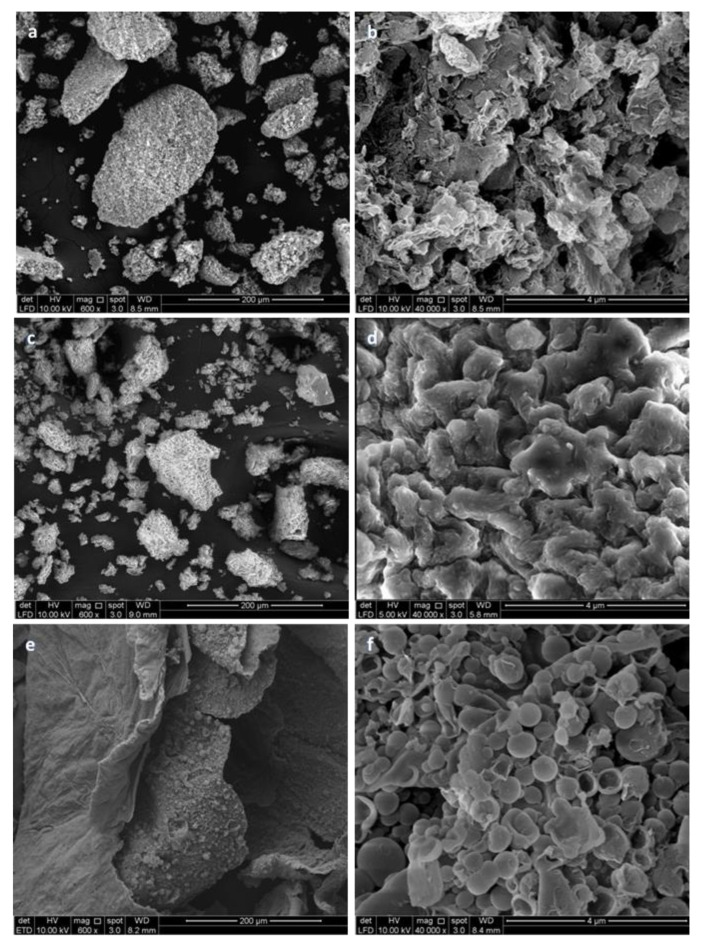
SEM micrographs of LP1 (**a**,**b**), LP2 (**c**,**d**), and BP (**e**,**f**) at 600× and 40,000×.

SEM analysis of surface morphology revealed that LP1 (Figure 3a,b) was more porous than the other two polymers. Although LP2 (Figure 3c,d) contains the same repeating units as LP1, its surface was smoother and less porous. This difference was due to different synthesis processes: monophasic oxidative polymerisation was utilised to produce LP1, whereas LP2 was obtained from interfacial polymerisation in two immiscible solvents in the Schotten-Baumann acylation reaction. Jayakannan, et al. [43] also found that monophasic oxidative synthesis of polyaniline dopant resulted in greater porosity than biphasic/interfacial polymerisation with vigorous stirring. LP2 also was produced by biphasic vigorous stirring and had a similar coral-like surface appearance as the polyaniline dopant.

BP has similar chemical structure to trimesic acid derivative disulfide cross-linked polymer as reported earlier [26]. Interfacial polymerisation method was adapted to produce microcapsules [44] as shown in Figure 3e,f. The polymerisation of BP occurred at the interface of two immiscible solvents or oil droplet surfaces [45,46]. Hence, after removal of the solvent in the core through evaporation, hollow microcapsules of BP were formed. In contrast, no micro/nanocapsules were produced for LP2, even though a similar interfacial polymerisation method was used to produce both BP and LP2. One possible explanation for this difference is that the pre-membrane of cross-linked BP may possess stronger and more stable mechanical properties during the process of miro/nanocapsule compared to the linear chain of LP2 [47,48]. In addition, the rate of hydrolysis of adipoyl chloride may be higher than the rate of growth of the micro/nanocapsule wall, thus causing the pre-membrane of LP2 to be highly fragile and easily broken through shearing energy [48,49].

Micro- and nanocapsules of BP ranged in size from 0.35 to 2.0 µm and were bigger than the trimesic acid-based nanoparticles (P10) (65 nm^−1^ µm) reported by Mat Yusuf et al. [26]. This difference could be because the different synthesis methodologies used (interfacial polymerisation and air oxidation thiol) require different conditions. Additionally, the size of the BP micro- and nanocapsules depended on the size of the emulsion that formed from water and DCM at the onset of the reaction. The droplet size of the emulsion was influenced by the applied shearing energy, and only stirring was used during the synthesis reaction of BP. Stirring provides lower shearing energy than other methods, such as sonication or homogenisation, and forms a mini-emulsion for synthesising nano-sized particle [50,51].

#### 3.2.5. EDX Micrograph and Elemental Mapping of Polymers

**Figure 4:**
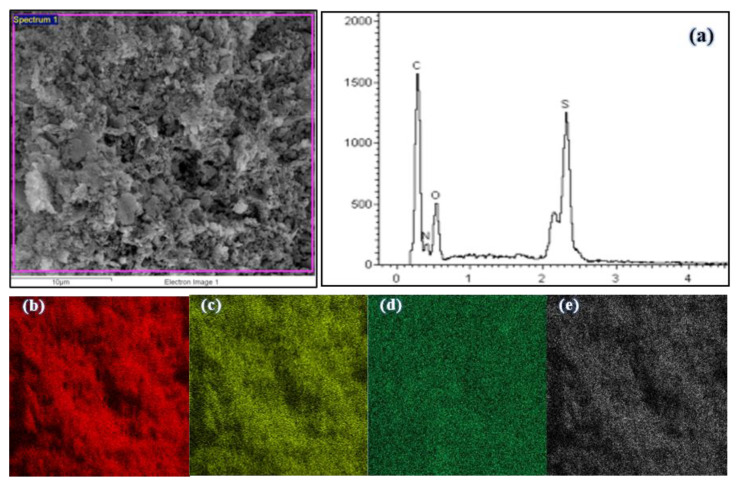
(**a**) EDX and elemental mapping for (**b**) carbon, (**c**) oxygen, (**d**) sulfur, and (**e**) nitrogen in LP1 at the magnification of 5000×.

**Figure 5:**
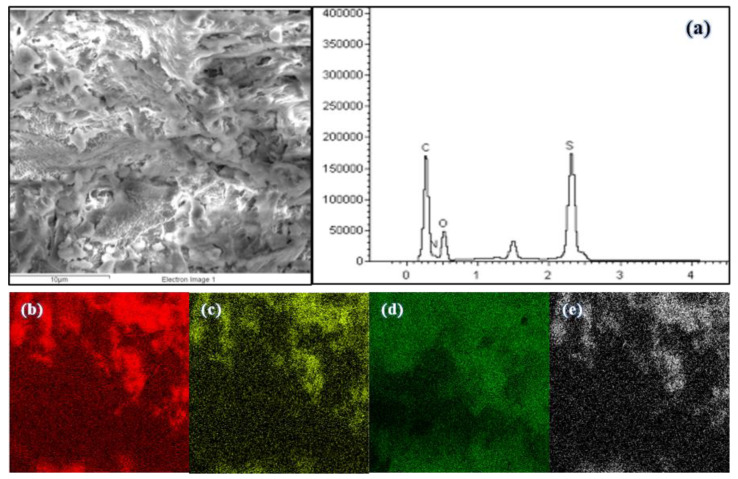
(**a**) EDX and elemental mapping for (**b**) carbon, (**c**) oxygen, (**d**) sulfur, and (**e**) nitrogen in LP2 at the magnification of 5000×.

**Figure 6:**
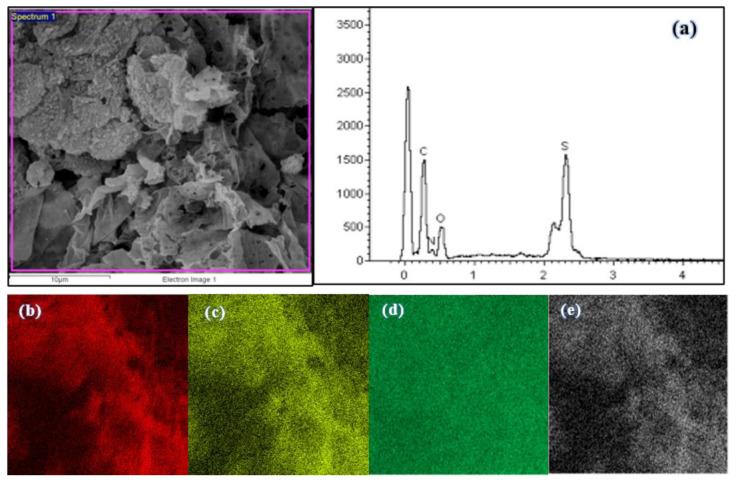
(**a**) EDX and elemental mapping for (**b**) carbon, (**c**) oxygen, (**d**) sulfur, and (**e**) nitrogen in BP at the magnification of 5000×.

The magnification of 5000× was selected for EDX mapping analysis of all three polymers. The EDX and mapping results showed equal distribution of carbon, oxygen, sulfur, and nitrogen for all polymers (Figure 4, Figure 5 and Figure 6), which suggested that polymerisation of all polymers occurred homogenously.

#### 3.2.6. Chemical Reduction of Disulfide Polymers

**Figure 7:**
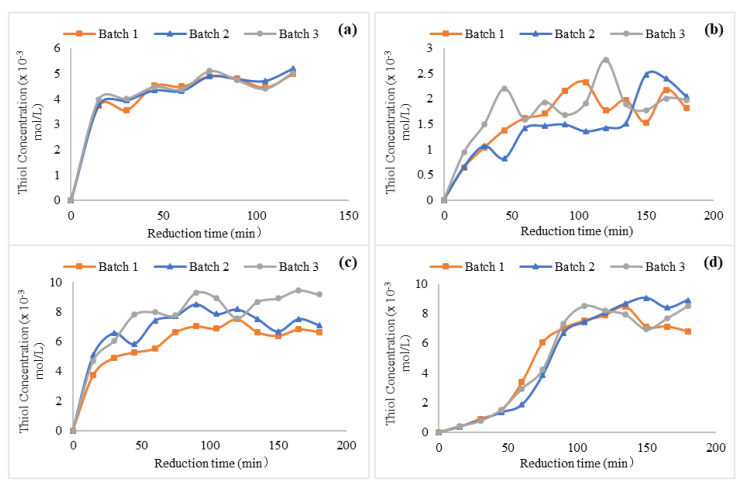
Chemical reduction of (**a**) cystamine dihydrochloride, (**b**) LP1, (**c**) LP2, and (**d**) BP.

The cross-linked polymer BP had the highest disulfide content (8.34 × 10^−3^ mol/L) followed by linear polymers LP2 (8.28 × 10^−3^ mol/L) and LP1 (1.99 × 10^−3^ mol/L) (Figure 7). BP had the highest values because the cross-linked bonds had more excess disulfide bonds in the repeating units compared to the linear bonds. Furthermore, BP had greater surface area compared to LP1 and LP2, which likely provides more accessible reduction sites [26]. LP1 and LP2 have similar repeating units, thus a similar trend in their reduction curves was expected. However, LP2 had a higher disulfide content than LP1, possibly due to different yields of final disulfide moieties. More disulfide moieties were available in LP2 because it was obtained directly from the polycondensation polymerisation of commercially available disulfide-containing cystamine and adipoyl chloride monomers. In contrast, the disulfide linkages in LP1 were obtained from oxidative polymerisation of 6 using DMSO in open air conditions. This may have resulted in oxidation of these dithiol monomers into sulfonic and sulfinic acid derivatives [31], thus subsequently preventing proper formation of disulfide linkages. Additionally, over-oxidation of thiols to thiolsulfinates and thiosulfonates may have occurred during the oxidative polymerisation of **6** [52].

#### 3.2.7. In Vitro Dissolution of Polymers

**Figure 8:**
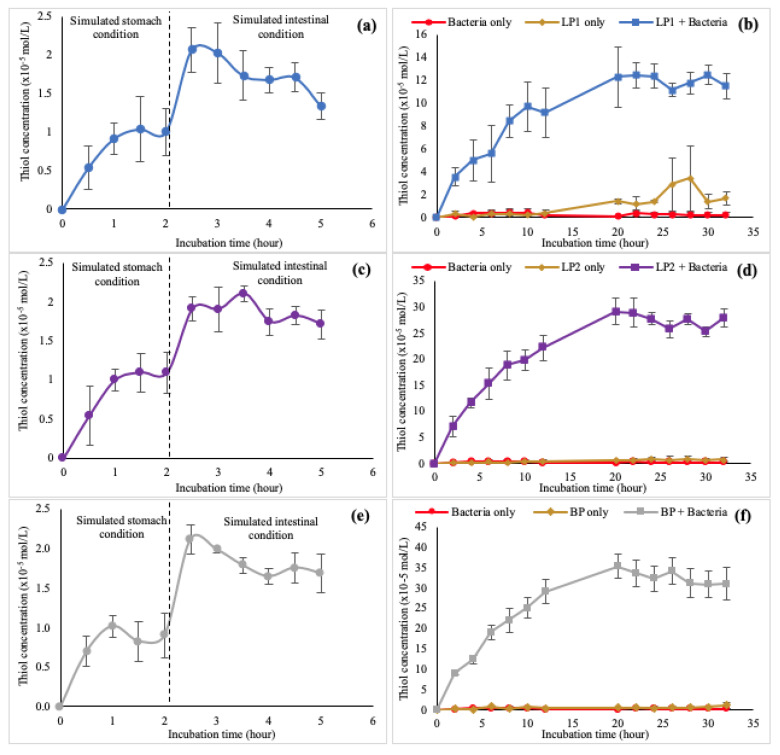
Free thiol concentration from dissolution of disulfide bonds in simulated gastric fluid and intestinal fluid (**a**) LP1, (**c**) LP2, and (**e**) BP, and simulated colon condition at 37 °C (**b**) LP1, (**d**) LP2, and (**f**) BP; Mean ± SD, n = 3.

LP1, LP2, and BP were incubated in the simulated gastric condition to study their stability in the acidic condition of the stomach. A lower thiol concentration was detected in the simulated gastric condition compared to the simulated colon condition (Table 2). After 2 h of incubation in the simulated gastric condition, polymers were recovered and reintroduced into the simulated intestinal condition for 3 h. The thiol concentration of each polymer detected in the simulated intestinal condition was higher than the thiol concentration in the simulated gastric condition (Figure 8). The values were relatively low compared to thiol concentrations of each polymer detected in the simulated colon condition (Table 2). Post-hoc Dunnet’s test (Table 2) revealed that all thiol concentrations detected in the simulated gastric and intestinal conditions for each polymer were significantly lower (*p* < 0.05) than the thiol concentration detected in the simulated colon condition in the presence of bacteria. Final thiol concentrations from the simulated gastric and intestinal conditions were more than 10 times lower than the final thiol concentration in the simulated colon condition in the presence of bacteria. This indicates that all polymers remained highly stable in the simulated gastric and intestinal conditions at pH 1.2 and 6.8 in the presence of pepsin and pancreatin.

The polymers were also incubated in the simulated colon in the presence of bacteria, *Lactobacillus*. *Lactobacillus* has shown a proportional increase in ulcerative colitis and Crohn’s disease patients [53,54]. The thiol concentration of each polymer incubated with bacteria increased within the first 12 h and eventually reached a plateau at the 20 h mark (Figure 8). The thiol concentrations of each polymer incubated in the simulated colon condition without bacteria and bacteria only were substantially lower than the thiol concentrations detected in the incubation of each polymer in the presence of bacteria, and the differences were statistically significant based on the post-hoc Dunnet’s test (Table 3). These results are consistent with previous reports by Lau and Lim [18] and Mat Yusuf et al. [26], who found that thiol concentrations in the simulated colon condition in the presence of bacteria were higher than in the simulated gastric and intestinal conditions as well as in the incubation of polymer without bacteria and bacteria only; however, they used different bacteria than the strain used in the present study. These results indicate that the reduction of disulfide polymers occurred because of the redox potential in the colon environment as effected by the presence of the anaerobic or facultative anaerobic bacteria [55] rather than being dependent on a reaction mediated by specific intestinal bacteria [56]. Also, from Figure 8, all the polymers took more than 20 h for full reduction in the colon, which is longer than the total transit time in the colon. This could be resolved by modifying and controlling the polymer coatings on drug (synthesis) formulation.

Despite having similar repeating units, the final thiol concentrations for LP1 (11.46 ± 1.08 × 10^−5^ mol/L) and LP2 (27.93 ± 1.65 × 10^−5^ mol/L) were different. This may be related to the lower availability of final disulfide moieties in LP1 as a result of oxidation during the fabrication process. Fewer disulfide moieties available in the reducing environment of the colon in the presence of *Lactobacillus* would result in a low final thiol concentration for LP1 compared to LP2. BP had the highest thiol concentration (31.00 ± 3.92 × 10^−5^ mol/L) among the three disulfide polymers. As a cross-linked polymer, BP has one additional thiol in each repeating unit compared to the linear polymers. Moreover, BP has a larger surface area than the than slab form polymers LP1 and LP2, making reduction easier [26]. However, the overall degradation trend and final thiol concentrations of LP2 and BP were quite similar. This is may be because linear polymers have a larger mesh size compared to cross-linked polymers after swelling, where solvation is prone to occur [57,58]. This may have enabled the bacteria to efficiently penetrate into the linear polymeric network of LP2. Furthermore, BP has a lower solubility due to its cross-linked nature, which means that it is more difficult for bacteria to permeate into the polymer to degrade the disulfide linkages of BP [59,60].

## 4. Conclusions

In the present study, two linear disulfide polymers (LP1 and LP2) with similar repeating units were successfully synthesised using different methods (oxidative polymerisation and interfacial polycondensation), and a cross-linked polymer (BP) also was successfully produced by interfacial polycondensation. The three polymers had different morphologies, with BP being the least porous and in the form of micro/nanocapsules due to the its cross-linked nature. All three synthesised polymers were stable in the harsh environments of the stomach and small intestine, and they were only reduced in the colon condition in the presence of bacteria. However, LP2 is preferable to LP1 because it required less time, used a more straightforward reaction, and had a better degradation profile than LP1. Although BP carries more thiol moieties than LP2, the reduction trends and final thiol concentrations were almost identical for these two polymers. Results of this study show that the degradation profile of the constructed polymers was affected by their morphology and the method of synthesis. More studies are needed to further validate the applicability of BP and LP2 as coating or matrix materials for colon-targeted drug delivery.

## Figures and Tables

**Table 1:** Solubility of LP1, LP2, and LP3.

Type of Solvent	Polymers		
	LP1	LP2	BP
Trifluoroacetic acid	√	√	√
Acetic acid	–	–	–
Formic acid	√	√	√
Acetone	–	–	–
Chloroform	–	–	–
Cyclohexane	–	–	–
Dichloromethane	–	–	–
Dimethylsulfoxide	–	–	–
Ethanol	–	–	–
Toluene	–	–	–
Water	–	–	–
Buffer system of gastric (without pepsin)	–	–	–
Buffer system of intestinal (without pancreatin)	–	–	–
Buffer system of colon (without *Lactobacillus*)	–	–	–

– = not soluble, √ = soluble, Temperature = 25 °C.

**Table 2:** Final thiol concentration (×10^−5^ mol/L) for LP1, LP2, and BP in (a) simulated gastric, (b) intestinal, and (c) colon (with bacteria) conditions; Mean ± SD. *n* = 3. The control sample contained the incubation medium for the simulated colon condition (polymer + bacteria).

Incubation Medium	LP1	LP2	BP
Gastric (a)	1.00 ± 0.30	0.90 ± 0.28	1.09 ± 0.25
Small intestinal (b)	1.34 ± 0.17	1.70 ± 0.18	1.68 ± 0.24
Colon (c)	11.47 ± 1.08	27.93 ± 1.65	31.00 ± 3.92
Statistical analysis	*p* < 0.05	*p* < 0.05	*p* < 0.05
Dunnett’s (significance)	a & ci b & ci	a & cii b & cii	a & ci b & ci

i = Dunnett’s T (2-sided) test, ii = Dunnett’s T3 test.

**Table 3:** Final thiol concentrations (×10^−5^ mol/L) for LP1, LP2, and BP and bacteria in different incubation media; Mean ± SD. *n* = 3. The control sample contained the incubation medium for the simulated colon condition (polymer + bacteria).

Incubation Medium	LP1	LP2	BP
Bacteria only (a)	0.22 ± 0.21	0.22 ± 0.21	0.22 ± 0.21
Polymer only (b)	1.67 ± 0.62	0.98 ± 0.52	1.11 ± 0.76
Polymer + bacteria (c)	11.46 ± 1.08	27.93 ± 1.65	31.00 ± 3.92
Statistical analysis	*p* < 0.05	*p* < 0.05	*p* < 0.05
Dunnett’s T (2-sided) (significance)	a & c b & c	a & c b & c	a & c b & c

## Supplementary Materials

The following are available online at https://www.mdpi.com/1999-4923/12/5/461/s1, Figure S1: ^1^H NMR of (triphenylmethyl)thioethylamine, **1**; Figure S2: ^13^C NMR of (triphenylmethyl)thioethylamine, **1**; Figure S3: DEPTQ 135° of (triphenylmethyl)thioethylamine, **1**; Figure S4: ^1^H NMR of *N*^1^,*N*^6^-bis(2-(tritylthio)ethyl)adipamide, **2**; Figure S5: ^13^C NMR of *N*^1^,*N*^6^-bis(2-(tritylthio)ethyl)adipamide, **2**; Figure S6: DEPTQ 135° of *N*^1^,*N*^6^-bis(2-(tritylthio)ethyl)adipamide, **2**; Figure S7: ^1^H NMR of [*N*^1^,*N*^6^-bis(2-mercaptoethyl)adipamide], **3**; Figure S8: ^13^C NMR of [*N*^1^*,N*^6^-bis(2-mercaptoethyl)adipamide], **3**; Figure S9: DEPTQ 135° of [*N*^1^,*N*^6^-bis(2-mercaptoethyl)adipamide], **3**; Figure S10: ^13^C CP/MAS Solid NMR of LP1; Figure S11: ^13^C CP/MAS Solid NMR of LP2; Figure S12: ^13^C CP/MAS Solid NMR of BP.
