# Supplementary material for: Redox-Sensitive Linear and Cross-Linked Cystamine-Based Polymers for Colon-Targeted Drug Delivery: Design, Synthesis, and Characterisation
Source: Pharmaceutics. 2020 May 18;12(5):461. doi: 10.3390/pharmaceutics12050461 (PMC7284438; doi:10.3390/pharmaceutics12050461)
Supplement: Supplementary file 1 [file pharmaceutics-12-00461-s001.pdf]

pharmaceutics

Supplementary Materials: Redox-Sensitive Linear and Cross-Linked Cystamine-Based Polymers for Colon-Targeted Drug Delivery: Design, Synthesis, and Characterisation

Yoke Mooi Ng, Siti Nur Aishah Mat Yusuf, Hock Ing Chiu and Vuanghao Lim

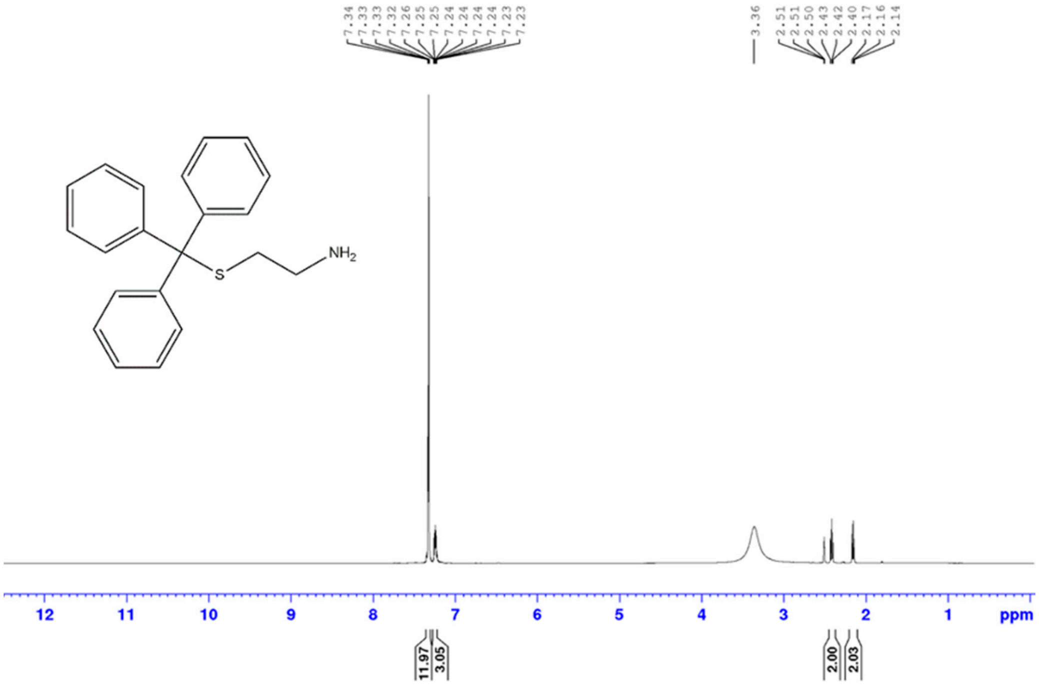

Figure S1. 1H NMR of (triphenylmethyl)thioethylamine, **1**.

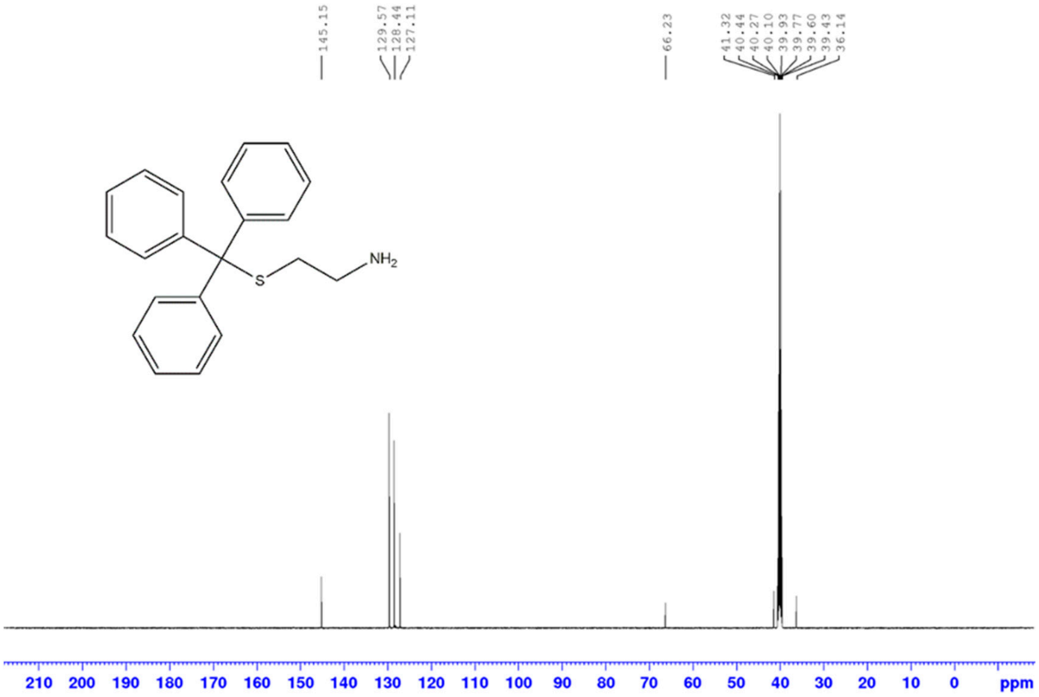

Figure S2. ^{13}C NMR of (triphenylmethyl)thioethylamine, **1**.

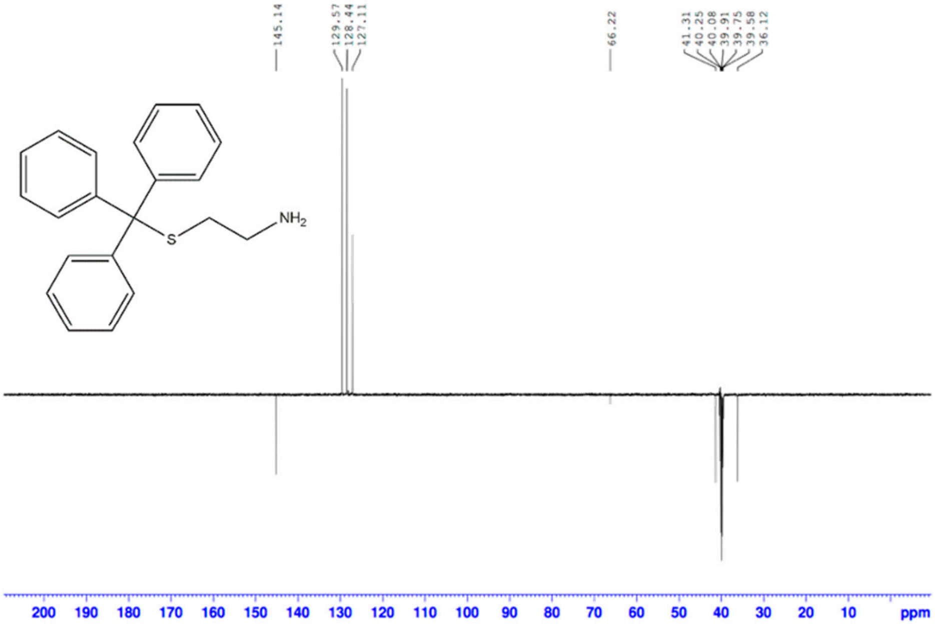

Figure S3. DEPTQ 135° of (triphenylmethyl)thioethylamine, **1**.

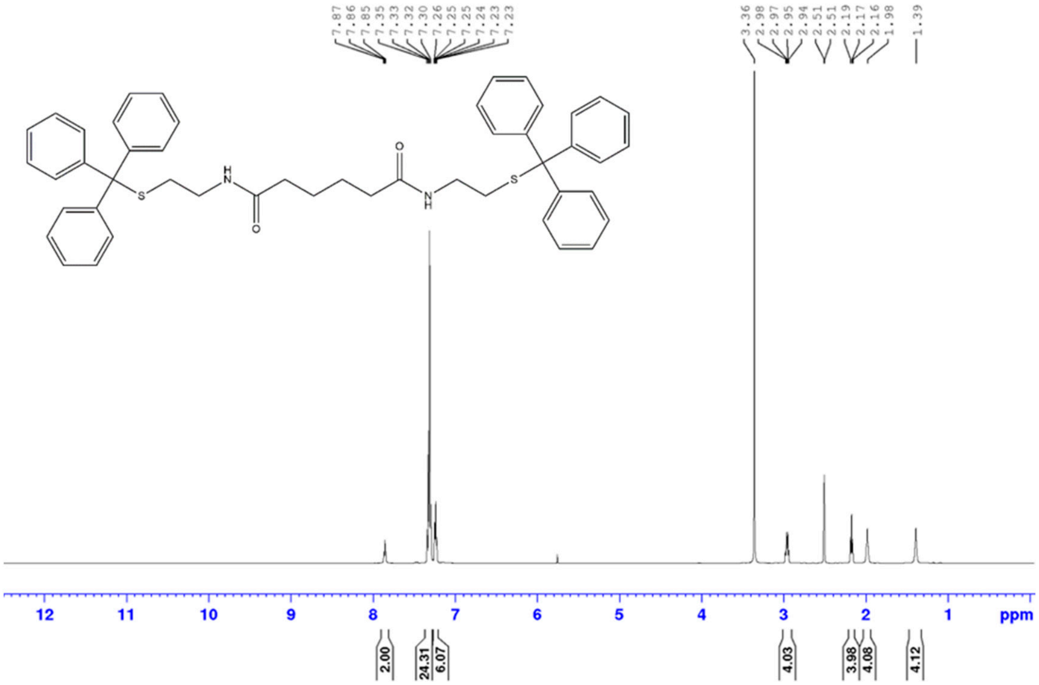

Figure S4. ^{1}H NMR of N^1,N^6-bis(2-(tritylthio)ethyl)adipamide, **2**.

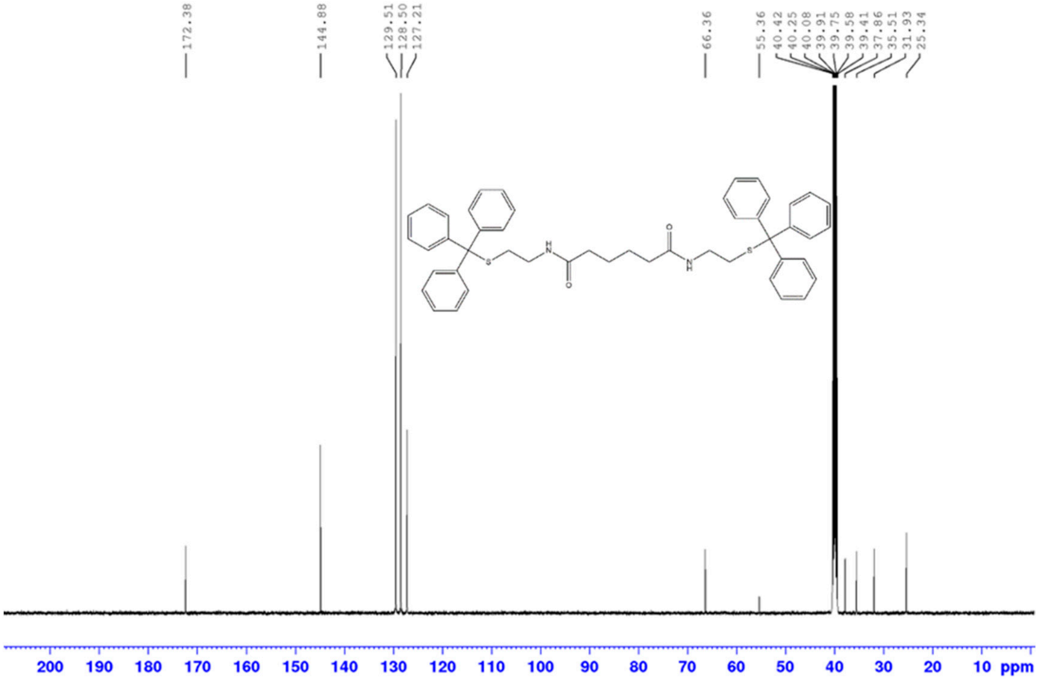

Figure S5. ^{13}C NMR of N^1,N^6-bis(2-(tritylthio)ethyl)adipamide, **2**.

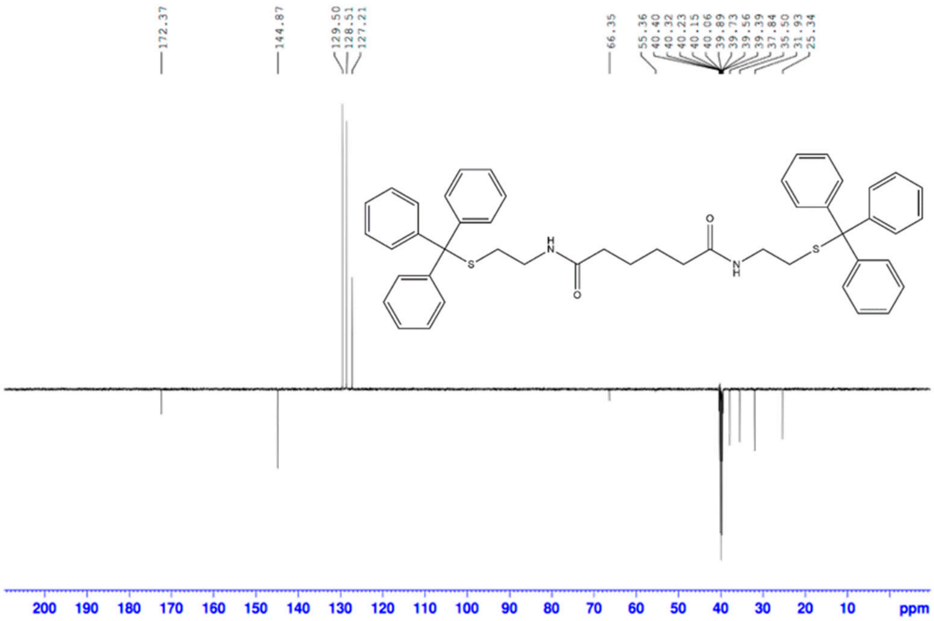

Figure S6. DEPTQ 135° of N^1,N^6-bis(2-(tritylthio)ethyl)adipamide, **2**.

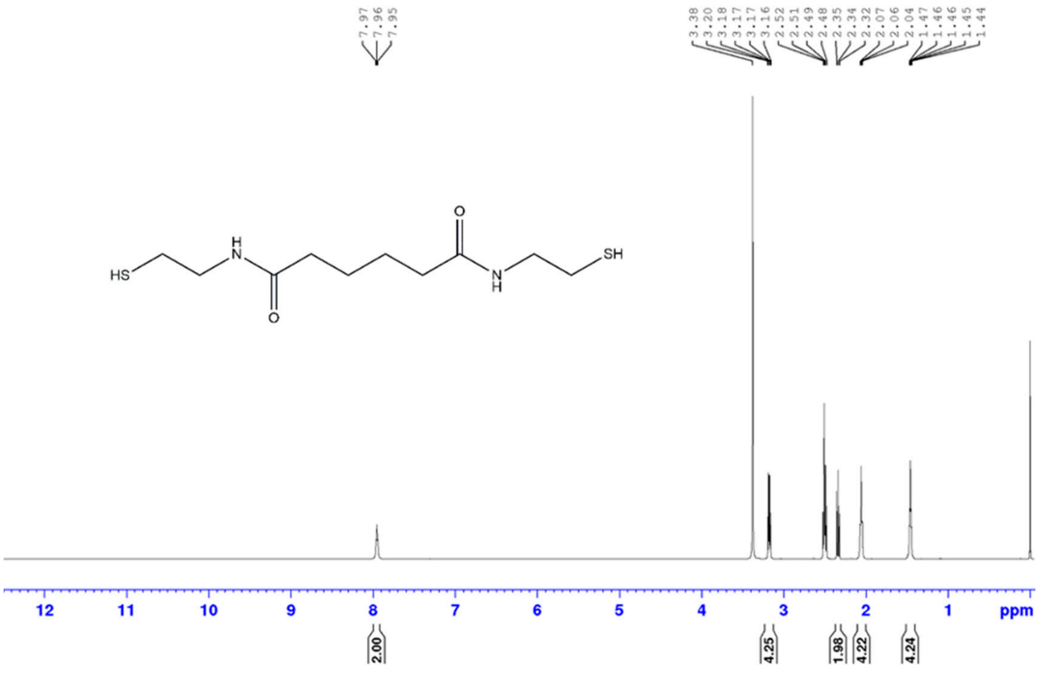

Figure S7. ^{1}H NMR of [N^1,N^6-bis(2-mercaptoethyl)adipamide], **3**.

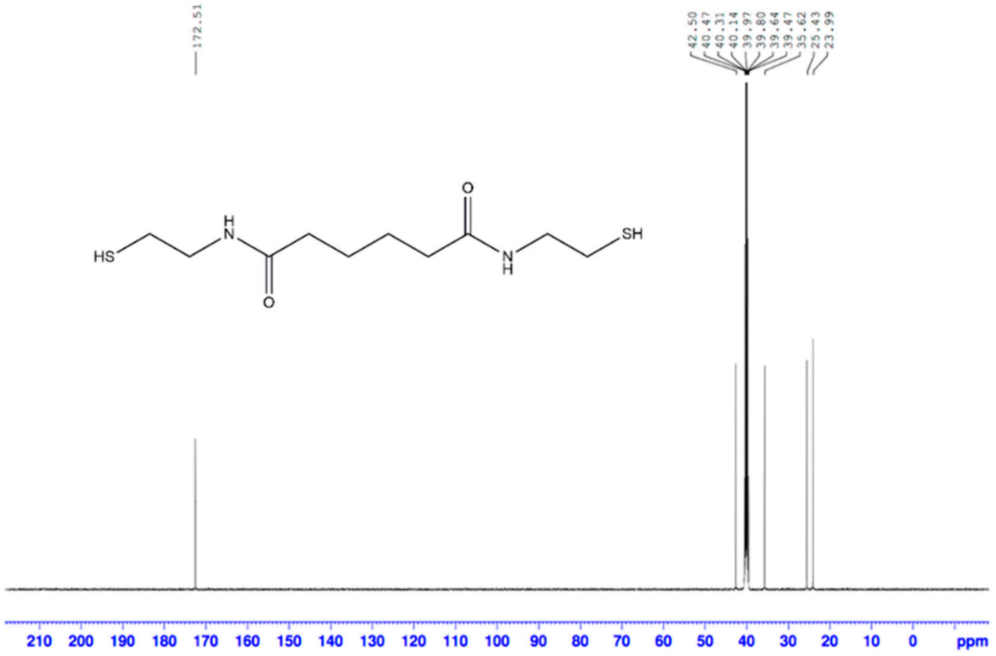

Figure S8. ^{13}C NMR of [*N*[1],*N*[6]-bis(2-mercaptoethyl)adipamide], **3.**

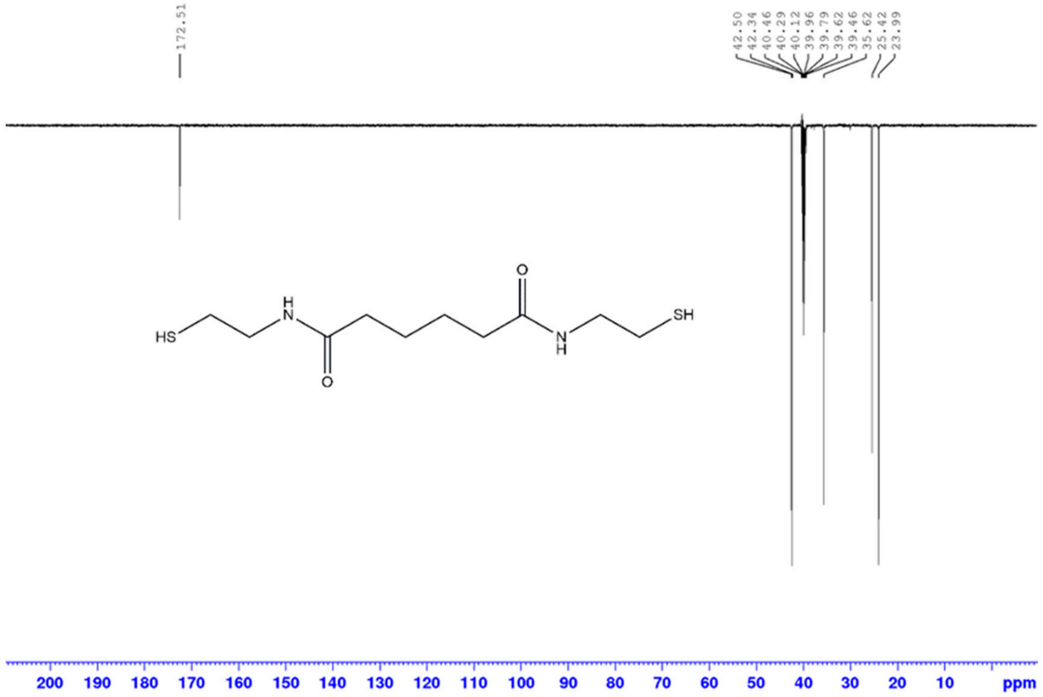

Figure S9. DEPTQ 135° of [*N*[1],*N*[6]-bis(2-mercaptoethyl)adipamide], **3**.

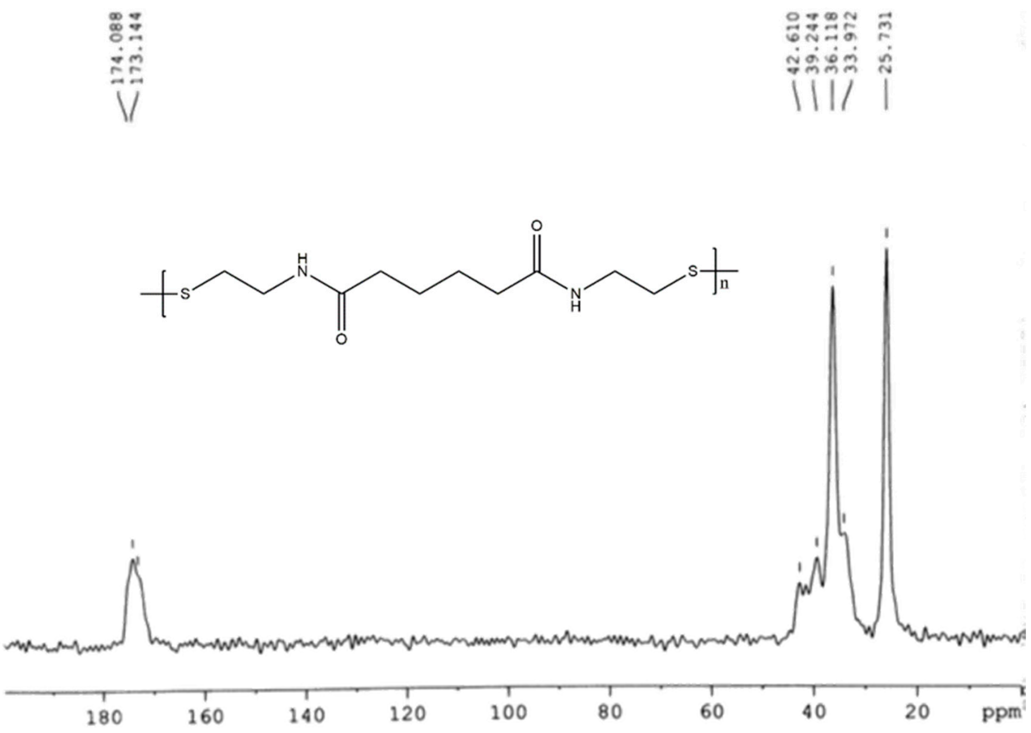

Figure S10. ^{13}C CP/MAS Solid NMR of LP1.

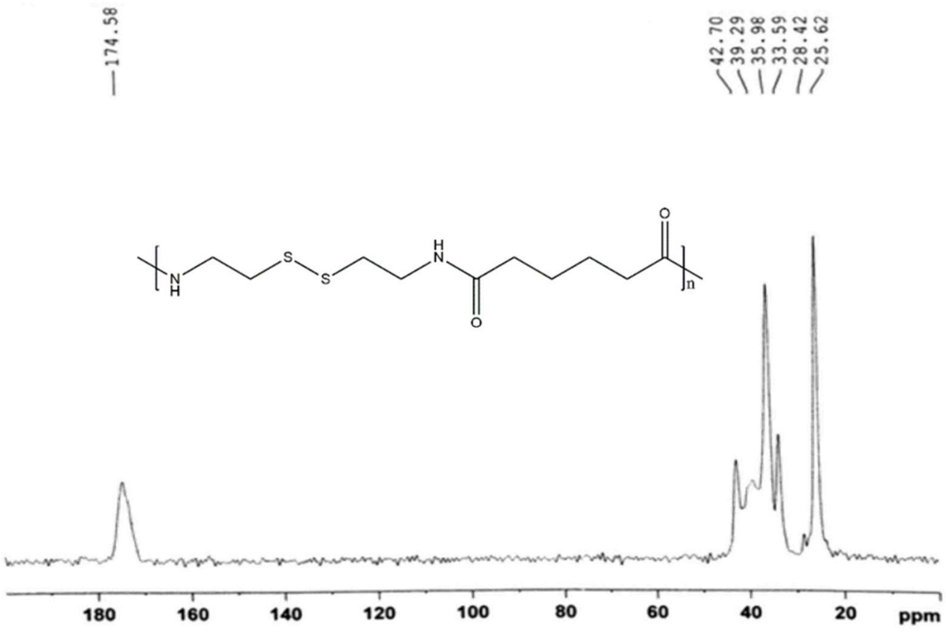

Figure S11. ^{13}C CP/MAS Solid NMR of LP2.

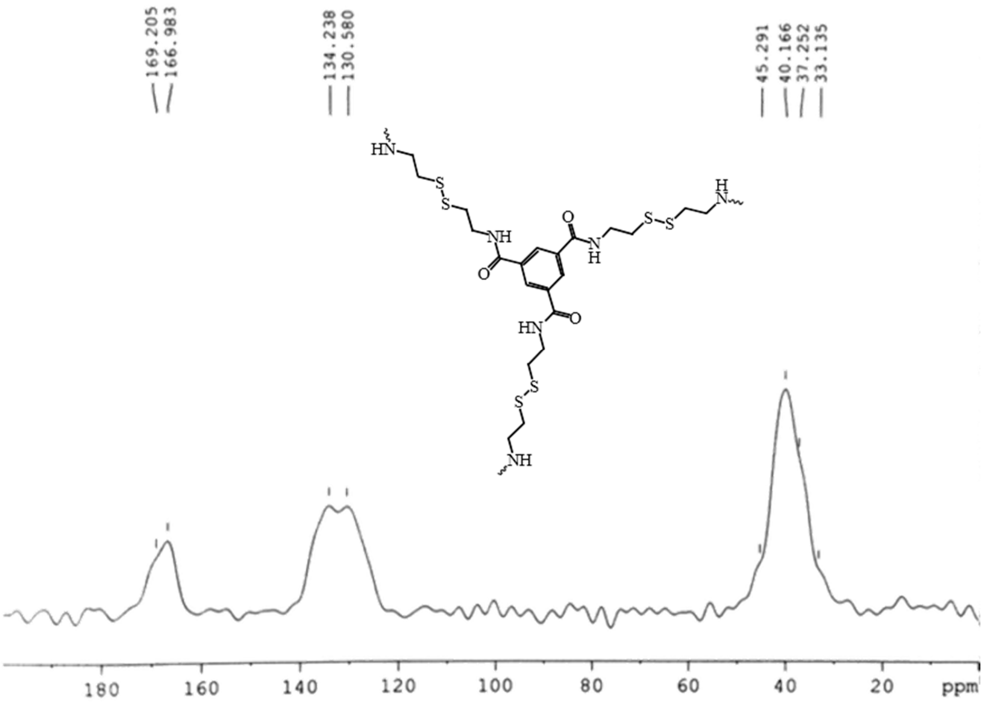

Figure S12. ^{13}C CP/MAS Solid NMR of BP.
